# Supplementary material for: The relationship of female physical attractiveness to body fatness
Source: PeerJ. 2015 Aug 25;3:e1155. doi: 10.7717/peerj.1155 (PMC4556148; doi:10.7717/peerj.1155)
Supplement: Table S1 [file peerj-03-1155-s001.docx]

| Image | Austria | | Lithuania | | UK | | China | | Iran | | Mauritius | | Kenya | | Morocco | | Nigeria | | |
| --- | --- | --- | --- | --- | --- | --- | --- | --- | --- | --- | --- | --- | --- | --- | --- | --- | --- | --- | --- |
|  | W | p | W | p | W | p | W | p | W | p | W | p | W | p | W | p | W | p |  |
| 1 | 1278 | 0.121 | 1194 | 0.369 | 2001 | 0.58 | 11123 | 0.056 | 10064 | 0.307 | 1413 | 0.687 | 1984 | 0.071 | 17595 | 0.544 | 9689 | 0.034 |  |
| 2 | 1156 | 0.143 | 1286 | 0.584 | 2021 | 0.703 | 9973 | 0.468 | 10670 | 0.463 | 1273 | 0.08 | 2166 | 0.546 | 17753 | 0.385 | 10692 | 0.346 |  |
| 3 | 1150 | 0.109 | 1221 | 0.639 | 2107 | 0.706 | 9379 | 0.037 | 10382 | 0.94 | 1430 | 0.5 | 1933 | 0.033 | 17322 | 0.876 | 10060 | 0.326 |  |
| 4 | 1198 | 0.568 | 1260 | 0.893 | 2061 | 0.982 | 9250 | 0.017 | 10065 | 0.308 | 1277 | 0.091 | 1960 | 0.05 | 15853 | 0.024 | 9673 | 0.03 |  |
| 5 | 1197 | 0.664 | 1190 | 0.336 | 2021 | 0.703 | 9703 | 0.179 | 10369 | 0.909 | 1377 | 0.895 | 2465 | 0.172 | 17979 | 0.215 | 11196 | 0.013 |  |
| 6 | 1203 | 0.775 | 1230 | 0.751 | 2175 | 0.33 | 11161 | 0.046 | 11342 | 0.005 | 1515 | 0.047 | 2515 | 0.09 | 17695 | 0.44 | 10029 | 0.282 |  |
| 7 | 1215 | 1.000 | 1235 | 0.805 | 1999 | 0.568 | 10795 | 0.248 | 10510 | 0.762 | 1372 | 0.834 | 2678 | 0.006 | 18139 | 0.133 | 10436 | 0.872 |  |
| 8 | 1193 | 0.593 | 1139 | 0.076 | 2133 | 0.547 | 10573 | 0.518 | 9894 | 0.127 | 1388 | 0.988 | 1890 | 0.015 | 17291 | 0.916 | 10160 | 0.499 |  |
| 9 | 1193 | 0.585 | 1210 | 0.52 | 1739 | 0.004 | 9806 | 0.267 | 10908 | 0.137 | 1447 | 0.352 | 2222 | 0.817 | 18108 | 0.146 | 11163 | 0.017 |  |
| 10 | 1246 | 0.456 | 1196 | 0.387 | 1884 | 0.112 | 9839 | 0.302 | 10760 | 0.295 | 1432 | 0.48 | 2407 | 0.325 | 16077 | 0.058 | 10986 | 0.066 |  |
| 11 | 1179 | 0.378 | 1238 | 0.843 | 2208 | 0.203 | 9523 | 0.079 | 11235 | 0.014 | 1369 | 0.798 | 2117 | 0.354 | 17370 | 0.813 | 9996 | 0.239 |  |
| 12 | 1232 | 0.682 | 1192 | 0.357 | 1983 | 0.476 | 10392 | 0.816 | 10251 | 0.642 | 1331 | 0.398 | 2444 | 0.221 | 18627 | 0.021 | 10654 | 0.407 |  |
| 13 | 1254 | 0.339 | 1203 | 0.45 | 2050 | 0.905 | 10497 | 0.637 | 9582 | 0.014 | 1458 | 0.271 | 2056 | 0.184 | 15788 | 0.018 | 9457 | 0.005 |  |
| 14 | *NA* | *NA* | 1248 | 0.975 | 2069 | 0.968 | 10676 | 0.378 | 10472 | 0.849 | 1352 | 0.598 | 2370 | 0.46 | 17241 | 0.981 | 10539 | 0.634 |  |
| 15 | 1229 | 0.747 | 1298 | 0.455 | 2119 | 0.632 | 10706 | 0.342 | 10625 | 0.518 | 1367 | 0.768 | 2225 | 0.833 | 18018 | 0.192 | 9804 | 0.078 |  |
| 16 | 1199 | 0.7 | 1282 | 0.628 | 2324 | 0.022 | 9738 | 0.206 | 10306 | 0.764 | 1244 | 0.028 | 2576 | 0.036 | 16676 | 0.364 | 10737 | 0.28 |  |
| 17 | 1231 | 0.7 | 1248 | 0.975 | 2160 | 0.397 | 10757 | 0.286 | 10033 | 0.265 | 1500 | 0.08 | 2063 | 0.199 | 16760 | 0.442 | 9775 | 0.064 |  |
| 18 | 1239 | 0.568 | 1322 | 0.263 | 2053 | 0.926 | 9571 | 0.099 | 10950 | 0.107 | 1272 | 0.078 | 2233 | 0.874 | 17486 | 0.669 | 10667 | 0.386 |  |
| 19 | 1231 | 0.709 | 1279 | 0.662 | 2055 | 0.94 | 11518 | 0.005 | 10625 | 0.518 | 1403 | 0.798 | 2481 | 0.141 | 17224 | 0.998 | 11092 | 0.03 |  |
| 20 | 1267 | 0.205 | 1309 | 0.361 | 2115 | 0.654 | 10870 | 0.184 | 10046 | 0.282 | 1366 | 0.756 | 2166 | 0.548 | 17069 | 0.796 | 10704 | 0.326 |  |
| 21 | 1160 | 0.172 | 1315 | 0.313 | 2020 | 0.7 | 10140 | 0.731 | 11164 | 0.024 | 1438 | 0.425 | 2305 | 0.759 | 17601 | 0.537 | 10706 | 0.323 |  |

**Table S1.** Mann-Whitney test results comparing the estimated physical attractiveness of 21 soft tissue DXA images by males and females in nine different populations (except Senegal)

**.**
