# Supplementary material for: The relationship of female physical attractiveness to body fatness
Source: PeerJ. 2015 Aug 25;3:e1155. doi: 10.7717/peerj.1155 (PMC4556148; doi:10.7717/peerj.1155)
Supplement: Table S2 [file peerj-03-1155-s002.docx]

**Table S2.** Multiple regression analyses of associations between body fatness, age and WHR and attractiveness.

Full analysis of variance tables and coefficients for significant effects.

1. Austria

| **Dependent variable** | **Df** | **Seq SS** | **Adj SS** | **Adj MS** | **F** | **P** |
| --- | --- | --- | --- | --- | --- | --- |
| Age | 1 | 14.528 | 1.263 | 1.263 | 2.54 | 0.129 |
| WHR | 1 | 19.790 | 5.754 | 5.754 | 11.59 | 0.003 |
| BF% | 1 | 55.247 | 55.247 | 55.247 | 111.28 | <0.001 |
| Residual | 17 | 8.440 | 8.440 | 0.496 |  |  |
| Total | 20 | 98.006 |  |  |  |  |
| S=0.704621 R-Sq=91.39% R-Sq(Adjusted)=89.87% | | | | | | |

|  | **Coefficients** | **S.E.** | **T** | **P** |
| --- | --- | --- | --- | --- |
| Intercept | 17.006 | 1.605 | 10.60 | <0.001 |
| Age | -0.02231 | 0.01398 | -1.59 | 0.129 |
| WHR | -7.083 | 2.081 | -.040 | 0.003 |
| BF% | -0.18076 | 0.01714 | -10.55 | <0.001 |

1. Lithuania

| **Dependent variable** | **Df** | **Seq SS** | **Adj SS** | **Adj MS** | **F** | **P** |
| --- | --- | --- | --- | --- | --- | --- |
| Age | 1 | 17.233 | 3.81 | 3.81 | 6.2 | 0.023 |
| WHR | 1 | 24.877 | 10.102 | 10.102 | 16.44 | 0.001 |
| BF% | 1 | 41.252 | 41.252 | 41.252 | 67.14 | <0.001 |
| Residual | 17 | 10.445 | 10.445 | 0.614 |  |  |
| Total | 20 | 93.808 |  |  |  |  |
| S=0.783861 R-Sq=88.87% R-Sq(Adjusted)=86.90% | | | | | | |

|  | **Coefficients** | **S.E.** | **T** | **P** |
| --- | --- | --- | --- | --- |
| Intercept | 18.369 | 1.785 | 10.29 | <0.001 |
| Age | -0.03874 | 0.01556 | -2.49 | 0.023 |
| WHR | -9.385 | 2.315 | -4.05 | 0.001 |
| BF% | -0.1562 | 0.01906 | -8.19 | <0.001 |

UK

| **Dependent variable** | **Df** | **Seq SS** | **Adj SS** | **Adj MS** | **F** | **P** |
| --- | --- | --- | --- | --- | --- | --- |
| Age | 1 | 13.418 | 2.403 | 2.403 | 4.09 | 0.059 |
| WHR | 1 | 30.367 | 13.263 | 13.263 | 22.58 | <0.001 |
| BF% | 1 | 43.393 | 43.393 | 43.393 | 73.89 | <0.001 |
| Residual | 17 | 9.983 | 9.983 | 0.587 |  |  |
| Total | 20 | 97.161 |  |  |  |  |
| S=0.766322 R-Sq=89.73% R-Sq(Adjusted) = 87.91% | | | | | | |

|  | **Coefficients** | **S.E.** | **T** | **P** |
| --- | --- | --- | --- | --- |
| Intercept | 19.244 | 1.745 | 11.03 | <0.001 |
| Age | -0.03077 | 0.01521 | -2.02 | 0.059 |
| WHR | -10.754 | 2.263 | -4.75 | <0.001 |
| BF% | -0.1602 | 0.01864 | -8.6 | <0.001 |

1. China

| **Dependent variable** | **Df** | **Seq SS** | **Adj SS** | **Adj MS** | **F** | **P** |
| --- | --- | --- | --- | --- | --- | --- |
| Age | 1 | 19.636 | 3.383 | 3.383 | 6.34 | 0.022 |
| WHR | 1 | 21.422 | 7.036 | 7.036 | 13.19 | 0.002 |
| BF% | 1 | 50.697 | 50.697 | 50.697 | 95.05 | <0.001 |
| Residual | 17 | 9.068 | 9.068 | 0.533 |  |  |
| Total | 20 | 100.823 |  |  |  |  |
| S=0.730337 R-Sq=91.01% R-Sq(Adjusted)=89.42% | | | | | | |


|  | **Coefficients** | **S.E.** | **T** | **P** |
| --- | --- | --- | --- | --- |
| Intercept | 17.746 | 1.663 | 10.67 | <0.001 |
| Age | -0.03651 | 0.0145 | -2.52 | 0.022 |
| WHR | -7.833 | 2.157 | -3.63 | 0.002 |
| BF% | -0.17316 | 0.01776 | -9.75 | <0.001 |

1. Iran

| **Dependent variable** | **Df** | **Seq SS** | **Adj SS** | **Adj MS** | **F** | **P** |
| --- | --- | --- | --- | --- | --- | --- |
| Age | 1 | 18.985 | 2.65 | 2.65 | 5.36 | 0.033 |
| WHR | 1 | 18.479 | 5.215 | 5.215 | 10.54 | 0.005 |
| BF% | 1 | 53.533 | 53.533 | 53.533 | 108.19 | <0.001 |
| Residual | 17 | 8.412 | 8.412 | 0.495 |  |  |
| Total | 20 | 99.409 |  |  |  |  |
| S=0.703424 R-Sq=91.54% R-Sq(Adjusted)=90.05% | | | | | | |

|  | **Coefficients** | **S.E.** | **T** | **P** |
| --- | --- | --- | --- | --- |
| Intercept | 16.982 | 1.602 | 10.6 | <0.001 |
| Age | -0.03231 | 0.01396 | -2.31 | 0.033 |
| WHR | -6.743 | 2.077 | -3.25 | 0.005 |
| BF% | -0.17794 | 0.01711 | -10.4 | <0.001 |

1. Mauritius

| **Dependent variable** | **Df** | **Seq SS** | **Adj SS** | **Adj MS** | **F** | **P** |
| --- | --- | --- | --- | --- | --- | --- |
| Age | 1 | 18.813 | 3.099 | 3.099 | 6.01 | 0.025 |
| WHR | 1 | 12.524 | 2.992 | 2.992 | 5.81 | **0.028** |
| BF% | 1 | 43.7 | 43.7 | 43.7 | 84.8 | <0.001 |
| Residual | 17 | 8.761 | 8.761 | 0.515 |  |  |
| Total | 20 | 83.798 |  |  |  |  |
| S=0.717886 R-Sq=89.54% R-Sq(Adjusted)=87.70% | | | | | | |


|  | **Coefficients** | **S.E.** | **T** | **P** |
| --- | --- | --- | --- | --- |
| Intercept | 15.294 | 1.635 | 9.35 | <0.001 |
| Age | -0.03494 | 0.01425 | -2.45 | 0.025 |
| WHR | -5.108 | 2.12 | -2.41 | 0.028 |
| BF% | -0.16077 | 0.01746 | -9.21 | <0.001 |

1. Kenya

| **Dependent variable** | **Df** | **Seq SS** | **Adj SS** | **Adj MS** | **F** | **P** |
| --- | --- | --- | --- | --- | --- | --- |
| Age | 1 | 14.019 | 3.167 | 3.167 | 7.26 | 0.015 |
| WHR | 1 | 33.872 | 16.005 | 16.005 | 36.71 | <0.001 |
| BF% | 1 | 40.365 | 40.365 | 40.365 | 92.58 | <0.001 |
| Residual | 17 | 7.412 | 7.412 | 0.436 |  |  |
| Total | 20 | 95.667 |  |  |  |  |
| S=0.660303 R-Sq=92.25% R-Sq(Adjusted)=90.89% | | | | | | |


|  | **Coefficients** | **S.E.** | **T** | **P** |
| --- | --- | --- | --- | --- |
| Intercept | 19.966 | 1.504 | 13.28 | <0.001 |
| Age | -0.03532 | 0.01311 | -2.7 | 0.015 |
| WHR | -11.813 | 1.95 | -6.06 | <0.001 |
| BF% | -0.15451 | 0.01606 | -9.62 | <0.001 |

1. Morocco

| **Dependent variable** | **Df** | **Seq SS** | **Adj SS** | **Adj MS** | **F** | **P** |
| --- | --- | --- | --- | --- | --- | --- |
| Age | 1 | 10.605 | 3.824 | 3.824 | 7.36 | 0.015 |
| WHR | 1 | 26.597 | 14.513 | 14.513 | 27.95 | <0.001 |
| BF% | 1 | 21.126 | 21.126 | 21.126 | 40.69 | <0.001 |
| Residual | 17 | 8.827 | 8.827 | 0.519 |  |  |
| Total | 20 | 67.155 |  |  |  |  |
| S=0.720573 R-Sq=86.86% R-Sq(Adjusted)=84.54% | | | | | | |

|  | **Coefficients** | **S.E.** | **T** | **P** |
| --- | --- | --- | --- | --- |
| Intercept | 18.215 | 1.641 | 11.1 | <0.001 |
| Age | -0.03881 | 0.0143 | -2.71 | 0.015 |
| WHR | -11.249 | 2.128 | -5.29 | <0.001 |
| BF% | -0.11178 | 0.01752 | -6.38 | <0.001 |

1. Nigeria

| **Dependent variable** | **Df** | **Seq SS** | **Adj SS** | **Adj MS** | **F** | **P** |
| --- | --- | --- | --- | --- | --- | --- |
| Age | 1 | 9.956 | 6.089 | 6.089 | 8.25 | 0.011 |
| WHR | 1 | 25.898 | 16.969 | 16.969 | 22.99 | <0.001 |
| BF% | 1 | 9.783 | 9.783 | 9.783 | 13.26 | 0.002 |
| Residual | 17 | 12.545 | 12.545 | 0.738 |  |  |
| Total | 20 | 58.182 |  |  |  |  |
| S=0.859051 R-Sq=78.44% R-Sq(Adjusted)=74.63% | | | | | | |


|  | **Coefficients** | **S.E.** | **T** | **P** |
| --- | --- | --- | --- | --- |
| Intercept | 17.99 | 1.957 | 9.19 | <0.001 |
| Age | -0.04897 | 0.01705 | -2.87 | 0.011 |
| WHR | -12.164 | 2.537 | -4.8 | <0.001 |
| BF% | -0.07607 | 0.02089 | -3.64 | 0.002 |

1. Senegal

| **Dependent variable** | **Df** | **Seq SS** | **Adj SS** | **Adj MS** | **F** | **P** |
| --- | --- | --- | --- | --- | --- | --- |
| Age | 1 | 7.517 | 3.554 | 3.554 | 11.70 | 0.003 |
| WHR | 1 | 15.180 | 8.849 | 8.849 | 29.12 | <0.001 |
| BF% | 1 | 9.577 | 9.577 | 9.577 | 31.52 | <0.001 |
| Residual | 17 | 5.165 | 5.165 | 0.304 |  |  |
| Total | 20 | 37.439 |  |  |  |  |
| S=0.551221 R-Sq=86.20% R-Sq(Adjusted)=83.77% | | | | | | |

|  | **Coefficients** | **S.E.** | **T** | **P** |
| --- | --- | --- | --- | --- |
| Intercept | 15.137 | 1.255 | 12.06 | <0.001 |
| Age | -0.03742 | 0.01094 | -3.42 | 0.003 |
| WHR | -8.784 | 1.628 | -5.40 | <0.001 |
| BF% | -0.07526 | 0.01341 | -5.61 | <0.001 |
