# Supplementary material for: The relationship of female physical attractiveness to body fatness
Source: PeerJ. 2015 Aug 25;3:e1155. doi: 10.7717/peerj.1155 (PMC4556148; doi:10.7717/peerj.1155)
Supplement: Figure S1 [file peerj-03-1155-s003.pdf]

Figure S1: Example images used in the study showing subjects with similar WHR but divergent Body fatness

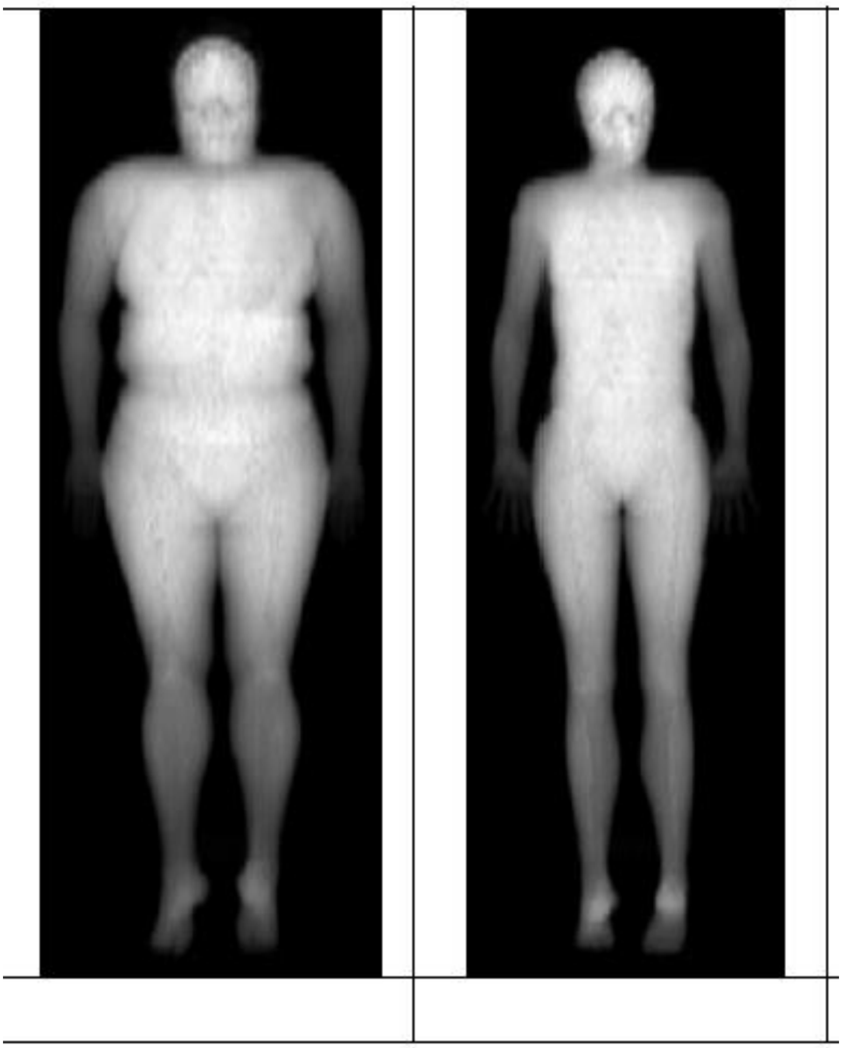

From Faries and Bartholomew (2012)
