# Supplementary figures and images for: The relationship of female physical attractiveness to body fatness
Source: PeerJ. 2015 Aug 25;3:e1155. doi: 10.7717/peerj.1155 (PMC4556148; doi:10.7717/peerj.1155)

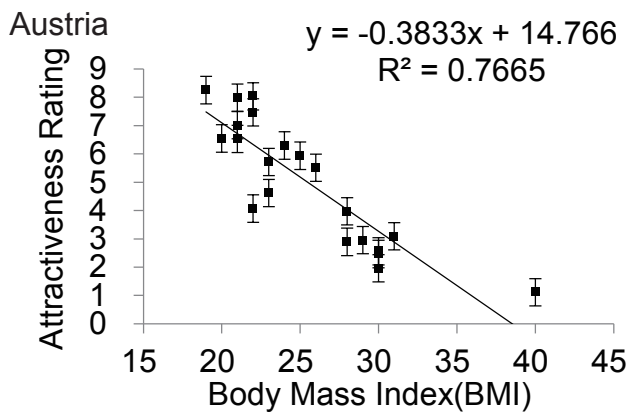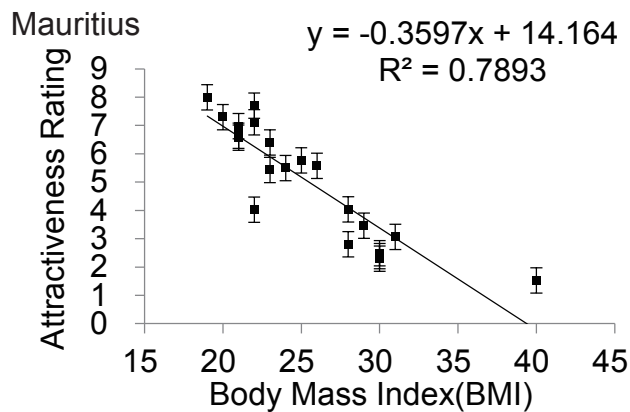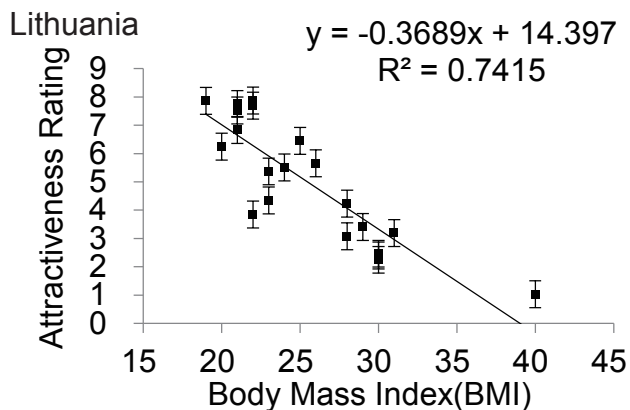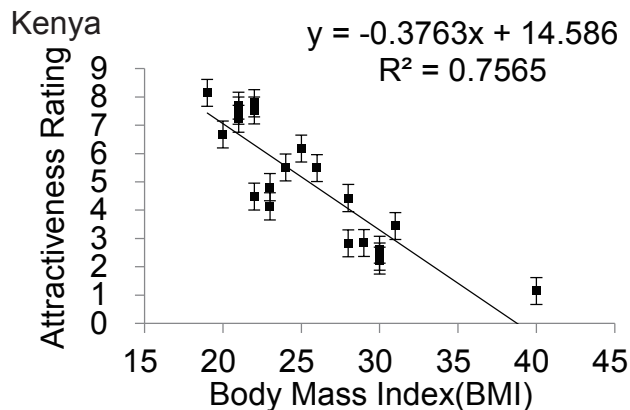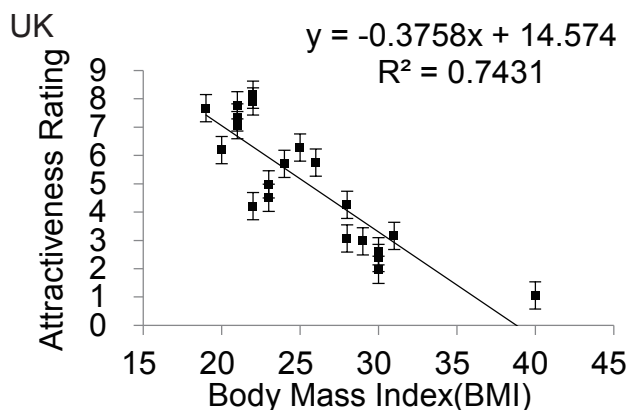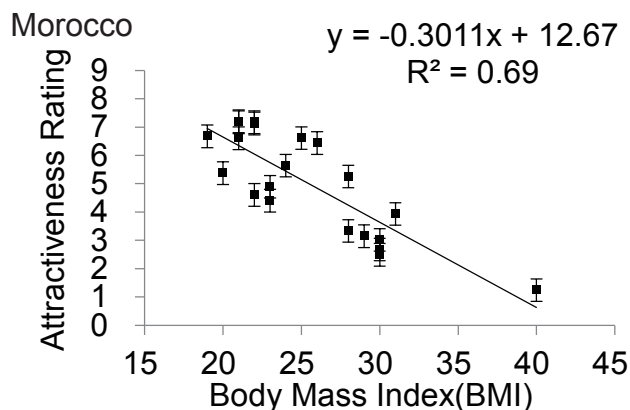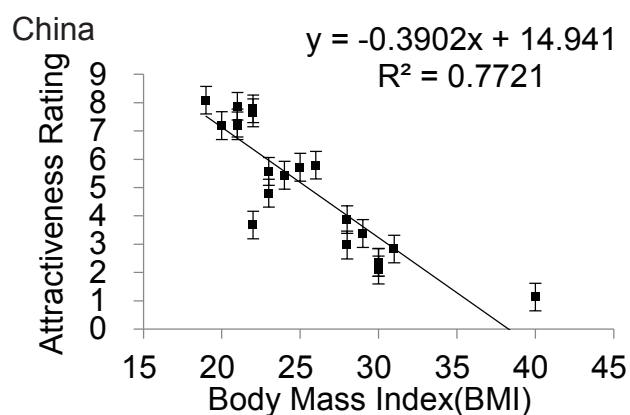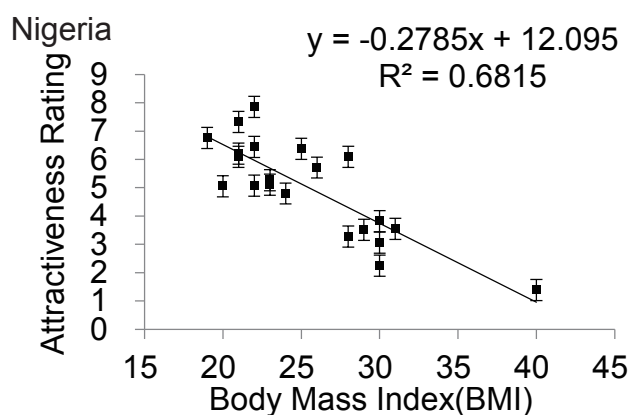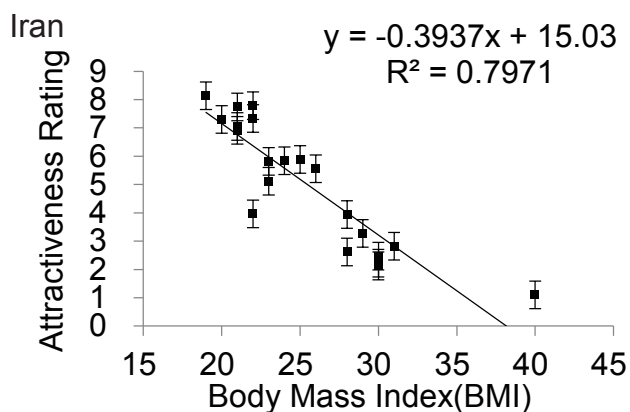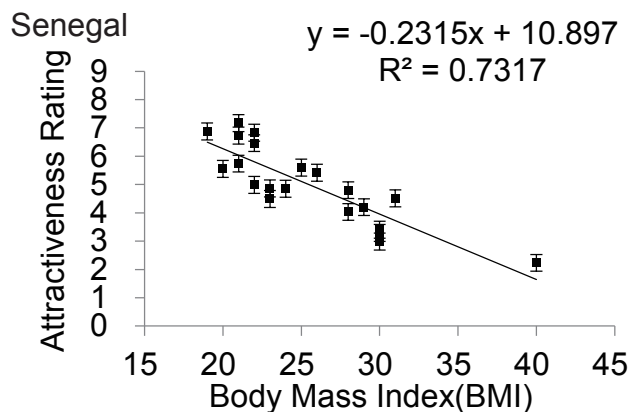

Supplement: Figure S2 — (Error bar referred to the standard error of both directions). [file peerj-03-1155-s004.pdf]
