# Supplementary material for: The relationship of female physical attractiveness to body fatness
Source: PeerJ. 2015 Aug 25;3:e1155. doi: 10.7717/peerj.1155 (PMC4556148; doi:10.7717/peerj.1155)
Supplement: Figure S3 — Infertility in relation to age of females measured as the live birth rate in females of given ages attempting to conceive, relative to the expectation if there was 100% fertility per 1,000 population. For ages below 20 the estimate is based on the live birth rate at age 20 multiplied by the annovulation rate since most children below 18 are not attempting to conceive. The fitted curve is the fourth order polynomial (refer to text for details). [file peerj-03-1155-s005.pdf]

Fgure S3: Infertility in relation to age of females measured as the live birth rate in females of given ages attempting to conceive, relative to the expectation if there was 100% fertility per 1000 population. For ages below 20 the estimate is based on the live birth rate at age 20 multiplied by the annovulation rate since most children below 18 are not attempting to conceive. The fitted curve is the fourth order polynomial (refer to text for details).

Infertility per 1000 Population attempting to conceive

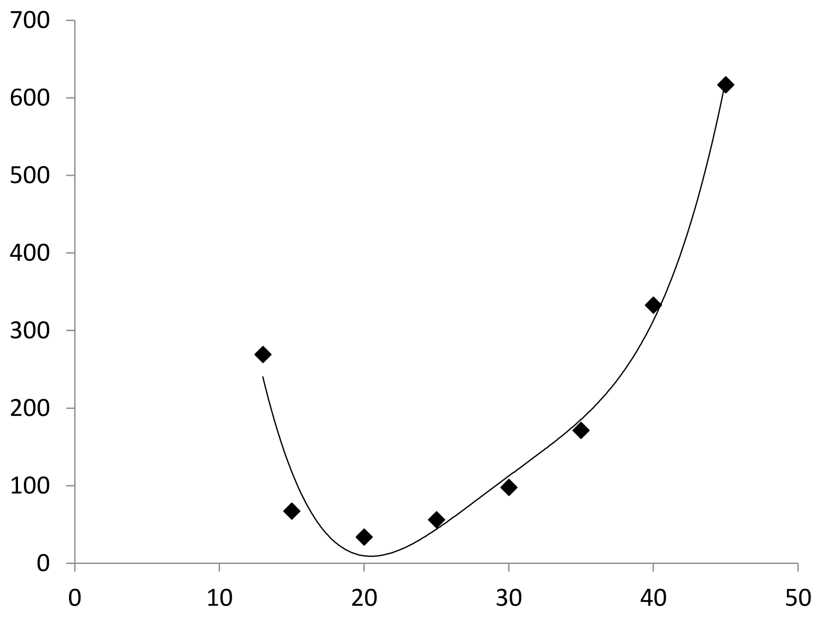

Age (years)
